# Development and Core Technologies for Intelligent SWaP^3^ Infrared Cameras: A Comprehensive Review and Analysis

**DOI:** 10.3390/s23094189

**Published:** 2023-04-22

**Authors:** Jingjie Jiao, Lixing Zhao, Wenhao Pan, Xiaoyan Li

**Affiliations:** 1 Hangzhou Institute for Advanced Study, University of Chinese Academy of Sciences, Hangzhou 310024, China; jiaojingjie21@mails.ucas.ac.cn (J.J.);; 2 University of Chinese Academy of Sciences, Beijing 100049, China; 3 State Key Laboratory of Infrared Physics, Shanghai Institute of Technical Physics, Chinese Academy of Sciences, Shanghai 200083, China

**Keywords:** infrared camera, intelligent, SWaP^3^

## Abstract

With the development of infrared detection and imaging technology, infrared cameras (IRCs) play an important role in many fields, such as military, industry, and civilian. Additionally, the requirements for the size, performance, and intelligence of IRCs are becoming more and more strict. Consequently, the associated research and development (R&D) of IRCs is gradually focused on the aspects of miniaturization, high performance, intelligence, low power consumption, and low cost, involving many frontier fields, including artificial intelligence, new materials, new optical systems, and electronics systems. In fact, there are continual studies on intelligent SWaP^3^ IRCs, but unfortunately, a systematic arrangement and analysis are lacking. Therefore, a systematical and comprehensive review for the developments and core technologies of the intelligent SWaP^3^ IRCs is really needed. In this paper, in terms of the aforementioned requirements, we conduct a review and analysis of current intelligent SWaP^3^ IRCs based on 90 literature and statistics in recent decades to provide the relevant developers with a helpful reference for facilitating the indicator optimization of intelligent SWaP^3^ IRCs with new developed technologies. We analyze the development of SWaP^3^ IRCs in the aspects of lightweight, miniaturization, low price, and high performance, including hyperspectral resolution, high spatial resolution, large field of view (FOV), and wide dynamic elaborately. Moreover, the development in low power consumption and intelligence is also discussed in detail. Additionally, we briefly summarize the primary applications of intelligent SWaP^3^ IRCs in military, scientific, and civil. Then, the core technologies comprising high-integration, lightweight, hyperspectral imaging (HSI), low-power consumption, as well as the realization of high performance such as high-resolution, high-frame, and wide-dynamic range of SWaP^3^ IRCs are discussed and analyzed in detail. Finally, we prospect for the intelligent SWaP^3^ IRCs that it is necessary to continuously expand the concept of SWaP^3^ by reliability, stability, extensibility, and safety. In addition, it is useful to embed cutting-edge technologies such as small pixel pitch array, multi-sensors fusion, and deploy intelligent algorithms to IRCs. Additionally, the improvement of the whole machine from multi-dimension such as chip, camera, and system is expected and needs to be taken more seriously. It is hoped that this paper can provide a reference for the R&D of intelligent SWaP^3^ IRCs in the future.

## 1. Introduction

**Figure 1:**
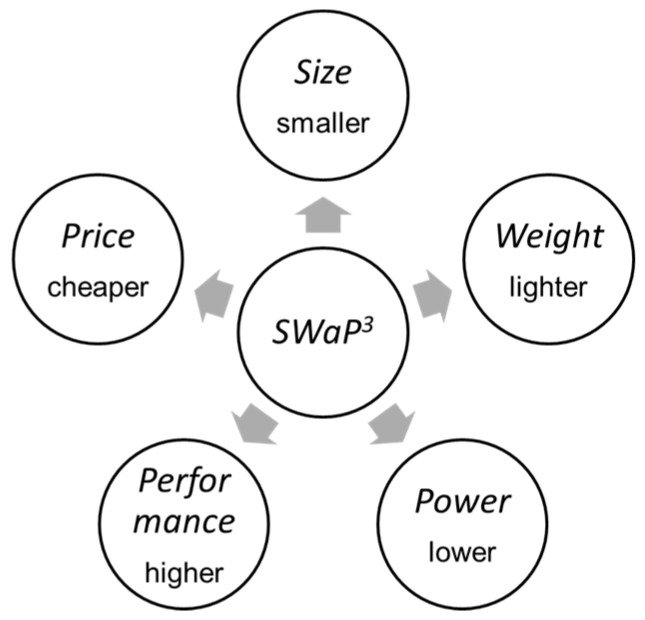
The concept diagram of SWaP^3^.

At present, due to the improvement of infrared detection and imaging technology, intelligent infrared detection and imaging products gradually tend to “SWAP”, namely size, weight, and power consumption. “SWAP” along with the high-performance (such as band, spectral information, signal-to-noise ratio, dynamic range, field of view (FOV), resolution, etc.) and the low-price constitute the new concept of “SWaP^3^” [1], as shown in Figure 1. SWaP^3^ reflects the current market variation tendency from the pursuit of ultimate performance to product availability, usability, manufacturability, and cost performance.

Intelligent SWaP^3^ cameras are now widely used in military fields, including military equipment, space remote sensing (RS) of key targets, space-sensitive target surveillance, planetary exploration, and in civil fields such as medical thermal imaging and temperature measurement, agricultural production, industrial security monitoring, forestry disaster prevention, and living entertainment [2]. The ultimate goal of current product development is to reduce the size, weight, power, and cost while meeting the performance requirements of the camera as much as possible. In terms of spaceborne applications, the microsatellite and CubeSat, with relatively lower weight, volume, and cost, shorter development cycles, and more widely applicable scenarios, have been rapidly developed in recent years. At the same time, with the rise of new materials and the continuous improvement of the detector, electronics, optical and other technologies, the corresponding payloads aboard the satellites above have also made breakthroughs, and micro miniaturization is gradually gaining attention [3]. Additionally, the miniaturization of imaging payloads undertaking critical tasks is one of the major difficulties during the micro miniaturization of payloads [4]. In ground scenarios, more and more applications tend to the edge scene, so small size, portability, low research and development (R&D) cost, mass production, and low power consumption have become the main demands nowadays. Additionally, the requirements for lightweight, power consumption, and cost of camera systems are getting higher and higher [5]. Therefore, the technical methods for each element of “SWaP^3^” are especially critical.

There are many universities, research institutes, and companies around the world focusing on the R&D of cameras led by SWaP^3^. In 2011, aiming at significantly reducing the size, weight, power, and production costs of uncooled cameras [6], DARPA initiated a Low-Cost Thermal Imager-Manufacturing (LCTI-M) program, in which DRS provided solutions for the LCTI-M program [7]. In particular, in terms of material, a silicon integration scheme was adopted. For the optical system, DRS eliminated most discrete components and used chip-level optics. In electronics, DRS is partitioned according to the electronic functions and uses state-of-the-art low-power FPGAs and memory devices. Additionally, in terms of packaging technology, a new 3D wafer-level package was adopted to achieve miniaturization. Finally, the solutions mentioned above facilitated the development of the SWaP^3^ module. BAE Systems developed the Stacked Modular Architecture High-Resolution Thermal (SMART) chip camera to reach the LCTI-M index requirements [8]. The SMART chip camera is manufactured using a wafer-on-wafer process. BAE Systems used a ROIC and an ASIC chip to reduce the size, which does not affect the FPA performance. Additionally, BAE Systems used wafer-level vacuum packaging to realize miniaturization. In addition, in recent years, DARPA also proposed a number of programs related to the R&D of intelligent SWaP^3^ infrared imaging equipment. The programs including the limits of thermal sensors (LOTS), focal arrays for curved infrared imagers (FOCII), and Blackjack all contain the objectives developing high-performance, small size, low weight, power and price detector chip, camera or satellite imaging payload. As shown in Table 1, in addition to DARPA, the organizations, agencies, and companies with strong capabilities in development of detector and camera including Leonardo DRS, BAE, ESA, NASA, Arizona Space Institute, FLIR, Sofradir, and Semi-Conductor Devices (SCD) have done a lot of works in the R&D of SWaP^3^ cameras.

At present, intelligent SWaP^3^ IRCs are gradually applied to edge scenarios with the extensive application of AI, embedded, and other technologies. Additionally, the cameras are increasingly focused on lightweight and low power consumption while improving their performance. However, it should be noted that the lightweight and miniaturization of the camera are contradictory to the improvement of the resolution, FOV, and other performance parameters. Therefore, how to find the right balance among these elements is a critical task for the development of intelligent SWaP^3^ IRCs. Currently, in almost all the scenarios, the overall development idea of new intelligent SWaP^3^ cameras is to realize miniaturization, low power consumption, and high reliability on the premise of achieving the target function and performance, but according to different application requirements, the emphasis is different. What is more, in terms of spaceborne applications, radiation resistance should also be considered.

Although many studies have been done on intelligent SWaP^3^ IRCs, there is still a lack of literature reviewing the development and core technologies of intelligent SWaP^3^ IRCs systematically and comprehensively. Therefore, this paper analyzes the specific situation of intelligent SWaP^3^ IRCs in depth. Our research mainly focuses on the studies and applications in the recent ten years, from 2010 to the present. Additionally, we prospect the development tendency of intelligent SWaP^3^ IRCs in multi-dimension to provide a reference for related research in the future.

## 2. Intelligent SWaP^3^ Camera

The development direction of IRCs fundamentally depends on the user’s requirements. The ultimate goal is to make the load as light and miniaturized as possible on the premise of achieving the application purpose and meeting the performance requirements with low power consumption and cost. The emphasis is different for various scenarios. Driven by the constantly evolving and iterative SWaP^3^, manufacturers have focused on some advanced directions such as miniaturization, lightweight, high performance (high-resolution, hyperspectral), and intelligence of IRCs. In applications such as surveillance globalization satellites and much strategic military equipment, the requirement for performance is unlimited. Additionally, the focus of scenarios dominated by high performance is to improve time resolution, spatial resolution, spectral resolution, and other core performance parameters such as sensitivity, frame rate, signal-to-noise ratio, as well as the degree of intelligence of the imaging devices. In these circumstances, form factor and power consumption are secondary considerations [11]. However, in civil fields, which are mainly edge scenarios such as security surveillance, industrial production, and medical assistance, engineers usually need to focus on all aspects of SWaP^3^ and find a right balance among performance, lightweight, miniaturization, power consumption, and cost.

This section analyzes the development of intelligent SWaP^3^ IRCs from various aspects of SWaP^3^, namely lightweight, miniaturization, low price, and high performance, including hyperspectral resolution, high spatial resolution, large FOV, wide dynamic, etc. We also analyze the development of cameras in the direction of low power consumption and intelligence in detail. Finally, this section introduces the applications of intelligent SWaP^3^ IRCs in military, scientific, and civil. Table 1 shows the technical indicators and core features of typical intelligent SWaP^3^ IRCs around the world, which are also covered and analyzed in this review.

### 2.1. Lightweight and Miniaturization

**Figure 2:**
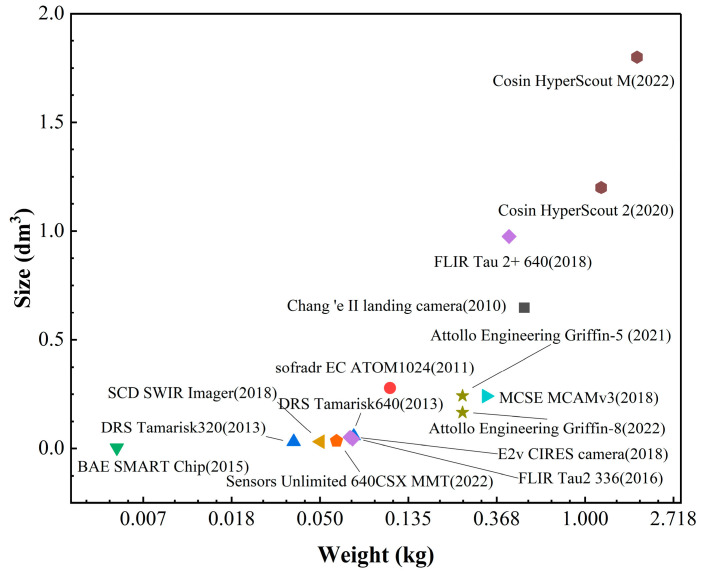
Comparison of the degree of lightweight and miniaturization of typical SWaP^3^ IR cameras developed by institutions and enterprises worldwide (the horizontal axis follows an Ln-type distribution).

In the past, engineers and developers were more concerned about reliability and high performance rather than lightweight, miniaturization, and low cost. As IRCs develop by leaps and bounds, people continue to expand their application scenarios in depth. So, in order to further promote and apply in civil, industrial, and other fields and make the camera more suitable for edge scenes, more and more manufacturers focus their attention on cost compression and equipment miniaturization, which becomes the main optimization goal in the camera design phase. Figure 2 shows the size and weight of typical SWaP^3^ IRCs developed by well-known organizations and companies worldwide in recent years. It can be clearly seen that the degree of lightweight and miniaturization has reached a very high level.

In the evolution of IRCs miniaturization, the detector, as the core component, is one of the key elements affecting the miniaturization. Infrared detectors have evolved from the first generation using optomechanical scanning to the current third generation of large-scale infrared focal plane arrays, which have higher performance in resolution and sensitivity [12]. Under this tendency, due to the progress and development of processing technic levels, applications of new materials, and integration technology, the number of components required to reach higher performance parameters has decreased rather than increased. At the same time, the area of electronic support required for each detector element has also been decreasing, and it varies from a few hundred square centimeters at the beginning to a few tens or even several square centimeters, which is small enough to support the normal operation of the detectors.

**Figure 3:**
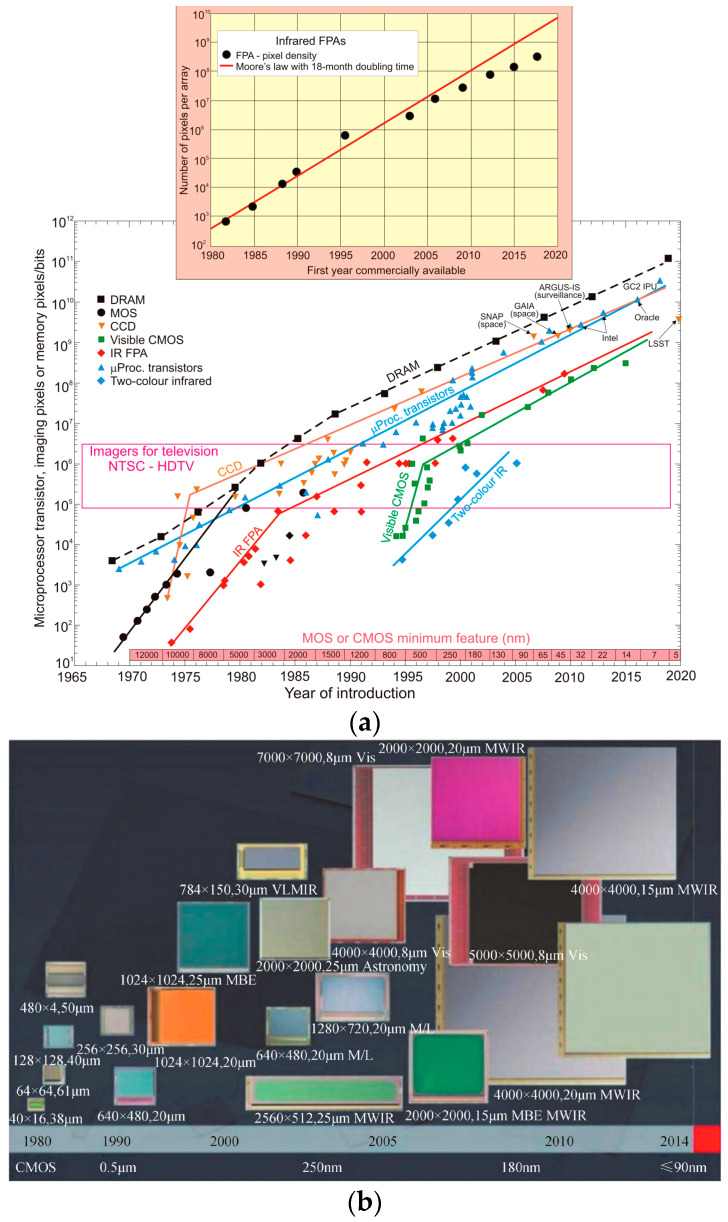
(**a**) Trends in various array sizes from 1965 to 2015 [13,14,15]; (Reproduced with permission from IOP, Reports on Progress in Physics; published by IOP, 2022) (**b**) The development of pixel pitch and array size for HgCdTe FPA in recent years at Raytheon Vision Systems [17,18].

Gradually, the resolution required for some scenes has increased significantly, so the pixel arrays have increased accordingly. Figure 3a shows the evolution of various array sizes from 1965 to 2015. The number of pixels of an infrared array has increased exponentially over a period of about 18 months, which has been keeping up with the development of integrated circuit technology and the ability of IC to read and process the array signals and display the image. It is inferred that the size of the infrared array will continue to increase in the future, but probably at a rate below the Moore’s Law trend [13,14,15]. At the same time, due to the continuous improvement of the detector development level, the pixel pitch is gradually reduced. Additionally, the overall area of the detector has not changed much. C. J. Alicandro et al. [16] found that with decreasing pixel size (pixel pitch) and increasing array sizes (number of pixels), the effective area of detectors remains relatively stable from low resolution 320 × 240 arrays to large scale 1024 × 768 arrays, i.e., their active detection area remains almost the same. In this case, production cost was relatively reduced, and the camera structure dimension was also controlled, which facilitated the development of lightweight and miniaturization. In short, reducing the pixel pitch can reduce the system size without changing the resolution. In other words, it can increase the number of focal plane pixels without changing the size area, thus increasing the resolution.

**Figure 4:**
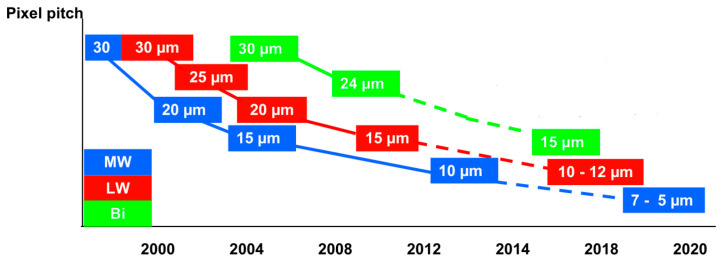
Development of pixel pitch of detectors by band for Sofradir in recent years [20]. (Reproduced with permission from SPIE; published by SPIE, 2011).

As a result, the main development trend of detector chips has been the continuous expansion of the array sizes and the reduction of the pixel size. In the development of the pixel size and array size of HgCdTe FPA at Raytheon Vision Systems in recent years, it can be seen in Figure 3b that the detector pixel size has been reduced from the initial 61 μm to 20 μm, 8 μm, and 5 μm today. In contrast, the array sizes have been expanding from the initial 40 × 16, 64 × 64 to 2 K × 2 K, 4 K × 4 K, and even 8 K × 8 K nowadays, which shows the decreased pixel pitch and the increased array size [17,18]. Similarly, Raytron Technology [19] has successfully developed infrared detector chips with pixel size in a sequence of 35 μm, 25 μm, 20 μm, 17 μm, 14 μm, 12 μm, 10 μm, 8 μm, …, and the array sizes’ released order is 160 × 120, 256 × 192, 384 × 288, 640 × 512, 1024 × 768, 1280 × 1024, 1920 × 1080, …. Figure 4 shows the change of detector pixel size for each band in recent years from French company Sofradir, which also confirms the trend of detector chip development.

Moreover, the pixel pitch of the detector can have an impact on other elements that directly determine the lightweight and miniaturization of the IRCs. As shown in a recent study by Attollo Engineering last year [21], the 5 μm pixel pitch process was applied to the MWIR detector, and the FPAs of 5 μm pixel pitch can directly translate into advantages of lightweight, miniaturization and low power consumption at the system level. With 5 μm pixel focal plane arrays, developers can achieve similar detection and recognition efficiency using a shorter focal length configuration. Additionally, the optical system is designed with more compact lenses, which makes the whole camera more lightweight and miniaturized. What is more, the reduced size of the focal plane itself and other components inside the Dewar reduces the pressure on the cooling equipment and the power consumption of the machine. In addition, the reduction of pixel pitch and shape of the focal plane can bring about a reduction in material costs. The study by Attollo Engineering shows that the use of small pixel pitch sensors can pave an important way for the implementation of SWaP^3^ IRCs, and this means it plays a positive role in all aspects of SWaP^3^. The development level of the detector chip determines the upper limit of the degree of lightweight and miniaturization for intelligent SWaP^3^ IRCs. The detector chip development has a profound impact on the detector itself, the optical system, the cooled equipment, the mechanical structure, materials, and many other aspects. Furthermore, reducing the pixel pitch and increasing the number of pixels can enable the detector to achieve better performance in scenarios such as identification tracking and long-distance long-term surveillance.

In conclusion, the level of detector development, process preparation, production, electronics, and optical system design has increased in recent years, and new materials have emerged continuously. In this trend, the lightweight, miniaturization, and cost control of intelligent SWaP^3^ cameras have been continuously broken through. As shown in Table 1, the whole size of the camera has been gradually reduced from the meter and decimeter level to the centimeter level, and the weight has been reduced from tens of kilograms to hundreds of grams or even tens of grams. As analyzed above, the development cost has been continuously compressed.

### 2.2. Low Price

The price of cameras is related to many elements, including the selection of materials, components, and chips, the design of electronics systems, optical systems, and packaging, and the optimization of cooling systems. Additionally, even some small elements, such as an inspection scheme for product quality, may affect the cost of cameras. Therefore, the price is an element that runs through the entire camera R&D process. In most cases, low price is achieved by cutting the cost of materials and components or optimizing the system design to reduce the amount of components usage, provided that these materials, components, and chips can achieve the desired target performance. For example, materials, preparation process, and production technology are the core elements that restrict the cost of intelligent SWaP^3^ IRCs. Table 2 shows the main materials used by worldwide famous detector development companies. Additionally, the materials are mainly HgCdTe, InGaAs, and VOx. Taking the selection of detector materials for the development of cameras in the SWIR band as an example, the materials used mainly include silica-based materials, Ge-based materials, and InGaAs. When considering commercial applications, the materials must be functionally feasible, technically reliable (theoretically proven), and commercially mature (practically applied) [22]. In this case, the material selection is focused on HgCdTe and InGaAs. In Patrick Oduor et al.’s study [23], it was mentioned that InGaAs detector wafers could be processed using MOCVD (metal–organic chemical vapor deposition) technology, which can process dozens of wafers at a time. As for HgCdTe, it can only grow one wafer at a time using MBE (molecular beam epitaxy) technology with a high production and processing cost. So, it is uncompetitive only from a cost perspective. In this study, a new FPA structure was adopted instead of the standard and traditional structure, which not only simplifies the fabrication process steps and reduces the process complexity and equipment cost but also reduces the pixel size to be able to spread in the submicron range. Additionally, it achieved a high yield of over 95%. Banpil Camera, developed based on the above technologies, is highly competitive in terms of price, with the whole machine design costing less than US$1000.

As mentioned above, one of the requirements of DARPA’s LCTI-M program is ‘Cost per Camera (fully functional) < US $500’. The limited budget available is often a tricky point that has to be taken into account for the SWaP^3^-constrained platform. By choosing a high-cost performance focal plane array and a low-cost package design, the SMART chip camera developed by BAE Systems achieved the expected price requirement of less than US $500. In addition, many other elements in the process of cameras’ R&D can affect the price. In terms of electronics and optical systems, optimizing the structure and layout to reduce the amount of component usage can further decrease costs. As for cooling IRCs, it is useful to optimize the design of the cooling system. Improving detector performance to increase the temperature condition for normal operation is another direction to reduce the need for cooling. Consequently, the cost of cooling to pay gets reduced. Moreover, the miniaturization of the above systems can further reduce the packaging cost. All of these dimensions can have an impact on the price of camera products, and the above are also several important approaches to cost optimization when producers consider actual camera product production.

### 2.3. High Performance

#### 2.3.1. High Spectral Resolution

The high spectral resolution is one of the features that SWaP^3^ IR cameras focus on, especially in applications such as RS. Hyperspectral RS imaging devices often have high spectral resolution and possess the capability of simultaneous spectral and image acquisition. The hyperspectral technology has wide and important applications in many fields such as earth science and applications (including geological exploration, soil, and water resources survey, agriculture and forestry surveillance, atmospheric environment monitoring, etc.), deep space exploration (Mars dust, lunar exploration, etc.), national defense and security (RS of key targets, military reconnaissance, etc.) [24,25].

**Figure 5:**
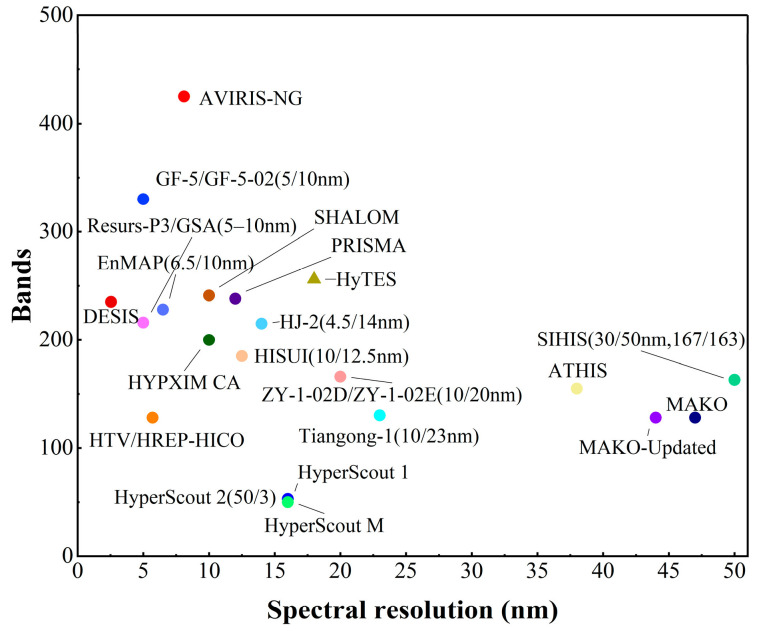
Comparison of core performance parameters of typical HSI devices in the world in recent years.

Compared with cameras using traditional imaging methods, hyperspectral cameras have the characteristics of multi-band, hyperspectral resolution, and large data volume. In terms of spectral resolution indicating the smallest wavelength interval that can be detected, hyperspectral imaging (HSI) is in the order of 10 nm with the number of bands ranging from tens to hundreds, while multispectral imaging is in the order of 100 nm with the number of bands ranging from a few to a dozen. This means that HSI has more bands and narrower spectral bands than multispectral imaging. Additionally, HSI can obtain more complex and accurate spectral feature information through higher spectral resolution, which makes the application scenarios more abundant. Furthermore, as shown in Table 3, the band range and the number of bands of hyperspectral cameras are increasing with technological breakthroughs. Figure 5 shows the two core parameters of spectral resolution and bands of typical HSI devices in recent years around the world. HSI technology for the infrared band usually covers a wide range of spectral bands from near-infrared to thermal infrared, and the suppression of infrared background radiation is particularly critical for HSI devices. When using infrared HSI systems, especially in the thermal infrared band, the peak wavelength of thermal radiation of the infrared detector itself overlaps with the wavelength of the targets, which makes it difficult to detect the signal of targets. Thus, it will affect the imaging performance of the instrument. To deal with the problem, designers often suppress background radiation through cryogenic cooling and infrared spectroscopy module. The spectral spectroscopy module is the core part of HSI systems, so the optical system design is bound to be affected by the background radiation suppression module. A good cryogenic optical system can effectively suppress the infrared background radiation, which can significantly reduce the NETD and thus improve the sensitivity of the system. Adopting reasonable measures of suppression of infrared background can achieve better imaging performance. Thus, in addition to the optical system, the cryogenically cooled module with the function of suppressing background radiation is the largest part of the volume and weight of HSI devices [26,27]. Due to the limitations of the instrument development level and unresolved technical problems, including IR fine spectroscopy and cryogenic optical technology aiming at background radiation suppression, the current IRCs with hyperspectral are mainly used in airborne applications. Additionally, applications for spaceborne monitoring are few. What is more, there are some problems, such as difficulty in data acquisition and long revisit cycles [28].

**Figure 6:**
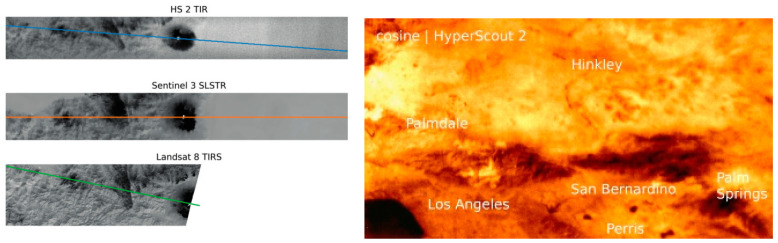
Thermal infrared band map taken by HyperScout 2 [31,32]. (The figure on the left shows the brightness temperature map over the volcano Mount Etna. The colored lines refer to the corresponding directions of the maps obtained by different loads.)

At present, the foreign company Cosine has accomplished a great breakthrough in the development of IRCs with hyperspectral. The HyperScout series developed by this company, a small intelligent hyperspectral camera dedicated to small satellites such as the CubeSat, is a typical representative of the cutting-edge hyperspectral intelligent SWaP^3^ IRCs [29]. Both models 2 and M in this series are compact enough to fit on very small satellite platforms and have relatively low cost and power consumption. The HyperScout 2 product has the ability to acquire spectral information in both VNIR and TIR bands [30], improving downlink efficiency through precise band alignment and increasing the ratio between useful and useless images acquired. HyperScout 2 achieves higher processing performance and lower power consumption. In addition, it realizes improving data processing capabilities for hyperspectral images by using Movidius processing board based on Intel’s new Myriad 2 AI chip, which provides high-performance edge computing for vision applications. As shown in Figure 6, in-orbit data from this device can be evaluated to be comparable to that obtained from the larger, higher cost and performance-oriented infrared HSI payloads carried by larger satellites.

Besides, adaptive spectral detection technology has also been applied in many scenarios requiring HSI information. The adaptive spectral detection technology achieves real-time processing of HSI data and enhances the adaptability to the environment by selecting and adjusting the number of spectral bands, wavelengths, and spectral resolution in real-time. In addition, it facilitates fast and accurate detection and determination of targets from complex backgrounds [33].

#### 2.3.2. High Spatial Resolution

Resolution is defined as the ability of a system to resolve details. For camera systems, high resolution means the ability to capture finer image details. What is more, spatial resolution for airborne or spaceborne cameras refers to the size of the smallest unit that can be distinguished in detail on a RS image, i.e., the ground size corresponding to a single pixel of the image. Imaging devices with high-resolution capabilities are widely used in many fields, such as RS monitoring, military reconnaissance, and civilian surveillance. Spatial resolution is one of the main parameters of IRCs. The development trend of spatial resolution is basically consistent with that of infrared detector array size. However, the spatial resolution of the detector used in the products constrained by SWaP^3^ is generally not high due to limitations of size, weight, power consumption, and other multiple conditions of the imaging system [34]. As Table 1 shows, in MWIR and LWIR bands, the spatial resolution is often from 0.3 Megapixels to 2 Megapixels. The specifications in common use are 640 × 512, 1280 × 1024, etc., such as Tamarisk 640 Camera, SMART Chip Camera, and FLIR Tau 2+. In SWIR and VNIR bands, the spatial resolution is often higher (this is because under the premise of the same size, the shorter the sensing wavelength, the smaller the area of the unit sensing element required. Thus, it can achieve higher spatial resolution) than that of MWIR and LWIR cameras. The spatial resolution can reach millions of pixels, with the most common specification being 1280 × 1024. Some cameras reach 4 Megapixels, such as the MCAMv3 camera developed by microcameras.space. Additionally, HyperScout 2, a hyperspectral IRC developed in recent years, has even reached a pixel scale of 4000 × 1850 in the VNIR band. The level of spatial resolution of SWaP^3^ IRCs is in the process of breakthroughs and some measures have been taken to reach high performance. The most direct ways to obtain high spatial resolution images are as follows: One is to increase the focal length of the optical system, but this makes the preparation of optical devices more difficult and costly and increases the weight and volume of the optical system [35,36]. The other is to reduce the pixel pitch of the detector, as mentioned above, but this will also cause problems such as reduced signal-to-noise ratio and reduced sensitivity. Besides, this method is limited by the level of development of small pixel-size detectors. Additionally, reducing the pixel size and increasing the array sizes will make the cost significantly higher. In addition to the above two methods, there are other methods such as multiple shift imaging and image enhancement to improve the image resolution by changing the imaging method or doing image processing [37,38,39,40,41].

To sum up, during the resolution enhancement, developers should comprehensively take many factors into account, such as resources, data processing capability, and noise characteristics, which need to be reasonably configured in combination with specific application scenarios. It should be noted that increasing the resolution of the camera will lead to a decrease in the signal-to-noise ratio. Furthermore, taking into account the resolution enhancement only will inevitably have a greater negative impact on other elements of SWaP^3^, such as a decrease in lightweight and a significant increase in cost.

#### 2.3.3. HDR, Large FOV, and Dynamic Windowing

Dynamic range is also one of the core elements of the performance parameters that SWaP^3^ focuses on. High dynamic range (HDR) is a kind of technique used to make the camera capture the characteristics of the image under very strong environmental contrast. In many scenarios where high and relatively low brightness exist simultaneously, dynamic switching occurs, such as under strong direct light, reflective environment, and accessing tunnels and caves; HDR can greatly enhance the user’s ability to obtain more DR, real-time image details and situation information [42,43,44]. The Banpil image sensor developed by Patrick Oduor et al. [23] has a dynamic range of >190 dB at the system level due to the ROIC and FPA technologies used in this sensor. So HDR of the Banpil image sensor allows the camera to acquire information about scenes with rapid changes in brightness, for example, from darkness to high brightness in the same frame or in successive frames. Moreover, it has a very high sensitivity.

IRCs have large FOV requirements in many military and civilian surveillance scenarios. Expanding the FOV can further increase the reconnaissance range, thereby enhancing the level of detection and target tracking and improving situation awareness capabilities. For example, in maritime surveillance scenarios, the U.S. Navy has assembled an experimental situation awareness system (SAWS) on aircraft carriers, which includes two SWaP^3^ IRCs placed at the bow and stern of the ship, respectively. The situation awareness camera group and multiple digital video recorders work together to achieve 360° all-round visual coverage for surveillance around the ship, which has great potential for applications in navigation, surveillance, identifying, and capturing targets.

Dynamic windowing (DW) is also one of the key technologies of intelligent, high-performance cameras, which mainly refers to the selection of the region of interest (ROI) during detector readout. By reducing the imaging area, DW can greatly increase the readout frame rate of the current region (other regions are with very low frame rates or are even completely static). So, the key is the removal of most of the uninteresting or irrelevant redundant information for localized very high frame rate output. The Banpil camera [23] has the feature of a variable frame rate. The frame of it can reach up to 1000 FPS at full resolution and up to 4500 fps in a 64 × 64 area when the window is focused on the target ROI, facilitating image information capture and high-speed data transmission in real-time.

### 2.4. Intelligence

With the in-depth research and application of many technologies, such as artificial intelligence, big data, and edge computing, IRCs are becoming more and more intelligent. The intelligence of SWaP^3^ cameras is often reflected in specific applications. Whether in spaceborne or ground-level scenarios, large centralized systems, or edge terminal devices, the degree of intelligence has been greatly improved.

UltraNav [45], developed by Astrobotic, is a typical representative of intelligent cameras in space-borne applications. As an intelligent camera system, it can realize computer vision-based tasks such as object recognition and tracking, state estimation, relative navigation, rendezvous and proximity operations (RPO), and deep space navigation. Additionally, UltraNav simplifies advanced and complex tasks such as Terrain Relative Navigation (TRN), SLAM, and 3D reconstruction. The algorithms for the aforementioned tasks are deployed on the system’s integrated dual-core CPU and FPGA, which supports energy-efficient and computationally efficient operations and allows for algorithm acceleration. UltraNav can realize TRN. In practice applications, it can pre-process the captured images and identify ROI. Then the ROI can be processed into signs. Furthermore, UltraNav can match these features to a database on a map, which can be used to estimate the absolute position and orientation of a spacecraft relative to the planets. It is also reconfigurable, with the ability to intelligently switch modes based on different application scenarios on demand. It weighs 2 kg and consumes less than 5 W, and the size is less than 1.5 U. So, UltraNav is a typical intelligent SWaP camera. Along with intelligent algorithms deployed on it, SWaP^3^ has become its main advantage. In resource-constrained scenarios where the performance of large payloads for complex missions is redundant, the above characteristics and advantages make it highly appropriate for scenarios requiring relatively low accuracy and other metrics. UltraNav can achieve simple image capture, image processing, or as a whole system for Guidance, navigation, and control (GN&C) solutions.

**Figure 7:**
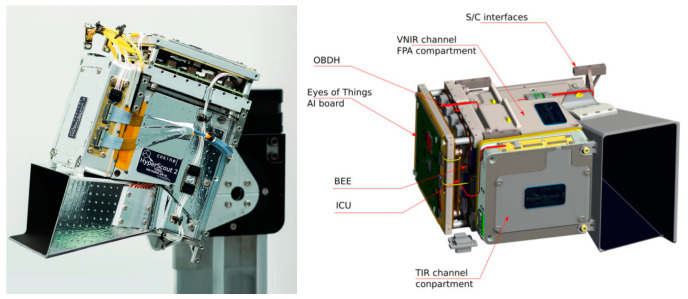
Physical map and structural diagram of HyperScout 2 [29].

The intelligence of the RS camera is well demonstrated on the HyperScout [29,30,46,47] series developed by Cosine. The electronics part integrates a dual-core CPU, GPU, and VPU for real-time on-orbit data processing and updating. As shown in Figure 7, Users can develop and run AI algorithms on HyperScout’s CPU-based OBDH (on-board data processing) or Myriad 2 VPU. Moreover, users can debug the BEE (back-end electronics) and OBDH subsystems with dual modes of acquisition and processing in orbit. The OBDH and BEE realize data transfer through the payload mass storage unit MMU. HyperScout is widely used in scenarios including urban heat island effects, oil spills, and natural disaster monitoring, including floods and fires. (Which is also covered in the synopsis of HyperScout in Table 1). What is more, HyperScout can autonomously calculate the distance between objects and the camera and their moving speed, which can be applied in fields such as capturing space debris. Different from previous HSI products, the most unique advantages of HyperScout are as follows: Firstly, HyperScout is extremely lightweight and miniaturized as a space-borne hyperspectral payload, which can be deployed on a smaller satellite platform with its characteristics with SWAP. It can observe changes in scenes at high speed and deliver processed information and coordinates in real time; satellites can achieve high revisit time on a global scale by deploying multiple units. Then, it is equipped with Intel Movidius Myriad 2 hardware accelerators for acceleration capability and robustness testing, by which the first CNN extrapolation on a satellite is realized to complete the identification, classification, and processing of cloud images. Through this system, HyperScout can process different levels of images in real-time and complete onboard data processing during space missions to reduce the amount of data that need to be sent back to Earth.

With the continuously increasing requirements of ground, airborne, and spaceborne platforms for lightweight and other functional indicators, many devices have already made a great breakthrough in weight and volume reduction. In the meanwhile, the degree of intelligence has also made a qualitative leap with the applications of AI. Throughout the development of intelligent IRCs, developers often integrate multiple functions, including image acquisition, data processing, data communication, and data storage, in a single device. Additionally, miniaturization, modularity, multi-functionality, and high reliability are often the core features of intelligent IRCs. During the R&D process, FPGA, DSP, etc., are often used as core processing components, and some products are equipped with new devices such as VPU. Moreover, they often have a unique machine vision processing scheme. Moreover, in order to further enhance the ease of use, convenience, and degree of intelligence, the products often provide supporting software that can be used for UI interaction to achieve image processing and editing [8].

### 2.5. Low Power Consumption

Low power consumption is one of the more difficult elements of SWaP^3^ to achieve, but it is extremely important in the design of practical applications. If the power consumption is too high or the cooling system is poor, it will directly affect the normal operation of the detector and, thus, the reliability. Therefore, in order to deploy in a wider range of applications, such as edge devices, it is important to take measures to reduce power consumption as much as possible while achieving the target performance requirements.

**Figure 8:**
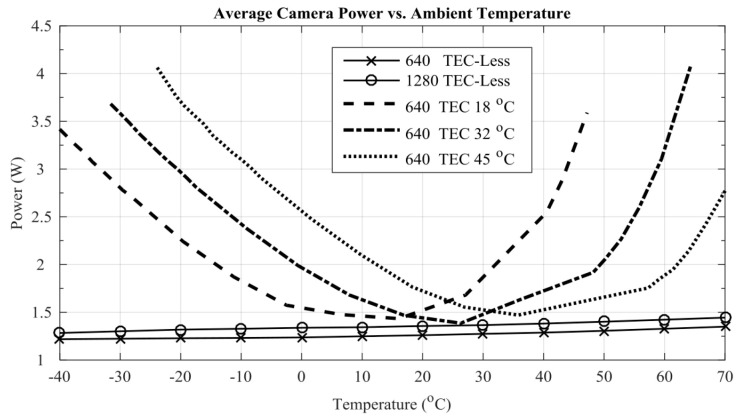
Variation of power with temperature for two resolution cameras with TEC and TEC-less [48]. (Reproduced with permission from SPIE; published by SPIE, 2016).

In the Banpil camera developed by Patrick Oduor et al. [23], although it possesses high sensitivity FPA and multifunctional, high-performance ROIC, it is still designed to achieve as low a power consumption as possible. The realization of low power consumption was to generate digital output through on-chip A/D conversion instead of additional components off-chip, through which the power consumption and space occupation were both reduced. As for cooled IRCs, in order to achieve a stable operating temperature, TEC (Thermoelectric Cooler) is often the largest part of power consumption. Jonathan Nazemi et al. designed and fabricated a SWIR imaging system without TEC [48]. They removed the TEC part, used current mirror pixels, and developed a compensation algorithm to achieve good day and night imaging performance in extreme temperature ranges. As shown in Figure 8, in the temperature range of −30~60 °C, the power consumption of all the cameras is in the range of 1.2~1.4 W. All of these studies took measures to replace or remove the components to reduce power consumption from the root. Additionally, they compensated for the corresponding performance loss through algorithms.

### 2.6. Applications

#### 2.6.1. Military and Scientific Field

The applications of intelligent SWaP^3^ cameras in the military and scientific fields can be divided into ground-level applications and space-borne applications. The former includes surveillance and reconnaissance, detection, identification, and tracking of key targets and military weapons development, and the latter consists of RS of sensitive objects, planetary and space exploration, and spaceborne surveillance. Many devices, including reconnaissance and surveillance cameras, are gradually developing in the direction of SWaP^3^. Additionally, various intelligent image processing and optimization algorithms are effectively deployed at the edge.

**Figure 9:**
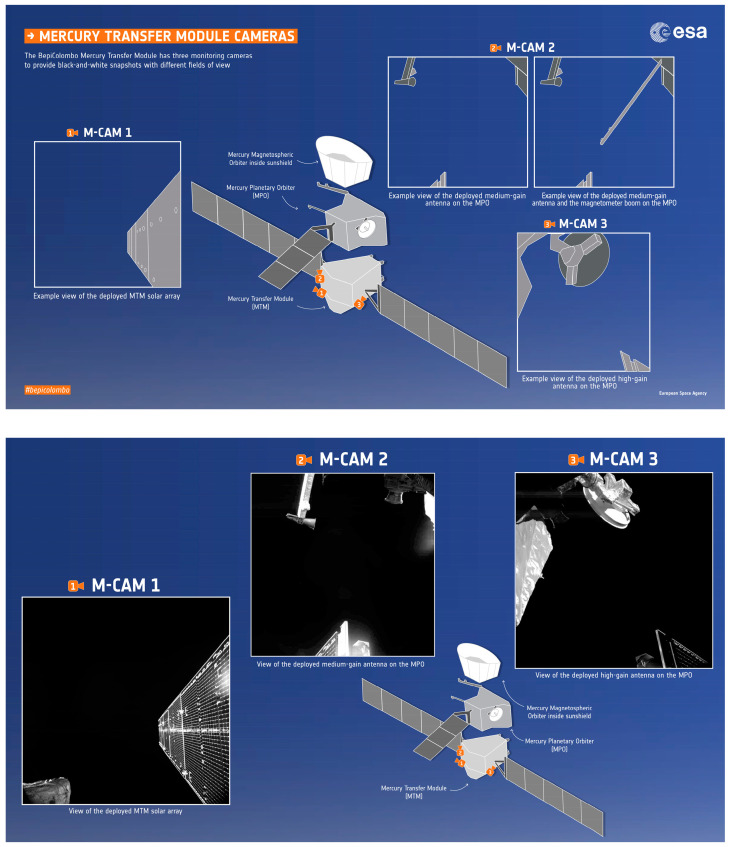
Deployment of Sentinel-1A’s cameras and images taken by the Monitoring Camera System [53,54].

In ground-level military systems, intelligent SWaP^3^ cameras are used in many scenarios, including military infrared vision enhancement equipment, maritime target surveillance, and target tracking. However, in this paper, we focus on the space military and scientific domain. The intelligent SWaP^3^ cameras are widely used in the monitoring and inspection of satellites themselves (including the monitoring of solar panels, antennas, and other key payloads) and in-orbit service with visual support (including the monitoring of critical events such as docking and separation of spacecraft) [3]. In the ESA’s BepiColombo program, the spacecraft carried a set of BepiColombo Selfie Cameras consisting of three MCAMs (micro-cameras) developed by MCSE, which are attached to a CAM Box (an interface box). The full name of the camera is MCAMv3 Digital Space Micro-Camera, and it is a multi-functional, modular, radiation-resistant SWaP^3^ module suitable for harsh environments. Furthermore, the system is responsible for capturing images of solar panel deployment during the LEOP (Launch and Early Orbit Phase) and the core module MTM (Mercury Transfer Module) MEPS (Micro-Satellite Electric Propulsion System) during the cruise phase. Additionally, the system can also be used for planetary surface photography [49,50,51]. As shown in Figure 9, in the European radar imaging satellite Sentinel-1A of the Copernicus program, MCSE also developed a Monitoring Camera System [52] with three miniature cameras and control processing units to monitor the deployment of solar panels and antennas during LEOP. Furthermore, one camera of the system is dedicated to the observation of the Earth with a weight of 110 g, power consumption of 1.8 W, and a large FOV of 70°.

What is more, in planetary research, intelligent SWaP^3^ cameras can be used to photograph the surface of planets or deep-space targets for research and analysis; in space debris detection and identification, SWaP^3^ cameras can detect foreign objects such as space debris and collect information from them to take avoidance measures or record them for research. In terms of space situation awareness, SWaP^3^ cameras can realize the identification and monitoring of various space targets such as high and low-orbit satellites (including active and abandoned satellites) and approaching satellites. Then the cameras obtain information and data from the above satellites, thus avoiding space collisions and conducting autonomous sensing and detection of in-orbit threats for avoidance or countermeasures. In regard to extraterrestrial planet detection, there are many intelligent SWaP^3^ IRCs developed for rover systems applying for exploration of Mars, the Moon, etc., which usually undertake tasks such as surface environment detection and analysis of planets [11,55,56].

#### 2.6.2. Civil Field

Intelligent SWaP^3^ cameras have wide applications in the civil field, including security monitoring, assisted driving, vehicle vision enhancement, and disaster prevention. For example, microthermal imaging cameras for monitoring abnormal areas of equipment are used in daily preventive testing. Additionally, in product R&D and electronics manufacturing, developers often use high-precision detectors to test and assess. Additionally, temperature guns are used for medical temperature measurement. Moreover, infrared imaging has the characteristics of long working distance and high imaging contrast, and it can work without external light sources, and IR imaging is less affected by abnormal weather. So, the IRC can be used as an auxiliary supplement to enhance the visual effect for high-performance and miniaturized monitoring edge equipment. Huawei “XMC” (extra, magic, credible) series [57] intelligent SWaP^3^ surveillance cameras use supplementary infrared light for auxiliary visual enhancement in addition to its own super starlight imaging capabilities. This series product can realize perimeter intrusion detection and behavior analysis.

Intelligent SWaP^3^ cameras are also widely used in agriculture. Unispectral’s Monarch is the first portable edge-end tunable spectral NIR SWaP camera in the world [58]. In the camera, Unispectral’s proprietary tunable Fabry–Perot filter (μFPF) and miniature IR camera module (including optics, image sensor, and controller) are integrated together on a 60 × 40 × 14.5 mm PCB. Furthermore, the module weighs 30 g in total and consumes less than 0.85 W, simplifying spectral imaging and eliminating the need for a bulky, complex, and expensive spectrometer. Additionally, it facilitates instant detection, monitorization, and classification. It has a wide range of prospects in agricultural scenarios, such as pest control, grading, and classification, as well as industrial and commercial scenarios.

In terms of medical scenarios, intelligent SWaP^3^ cameras can be used to detect body temperature in a non-contact, rapid, and non-hazardous way, which is suitable for the screening of large numbers of people. In recent years, infrared thermography and TIR products have been widely used during epidemics. In addition, intelligent SWaP^3^ IRCs can be used to assist in surgeries or medical examinations for achieving intelligent medical treatment along with many devices equipped with high technology. For example, the infrared radiation of the human body can be captured and converted into a dedicated thermogram by a small and light camera, thus facilitating the localization and detection of lesions, which can provide a basis for clinical diagnosis. Furthermore, the smaller size and lower weight of SWaP^3^ cameras can be used in more complex and difficult medical scenarios such as gastrointestinal examinations. The SWaP^3^ cameras can achieve more clear monitoring of lesions and accomplish more reliable and on-time localization of difficult diseases such as cancer. Then, doctors can save lives by taking medical measures or doing operative planning in advance.

Moreover, HSI devices are wildly used in medical fields. HSI can acquire both two-dimensional spatial information and one-dimensional spectral information of an object with a high spectral resolution, thus enabling the identification of various pathological conditions. It can provide a wealth of spectral information about patients’ tissue samples or disease sites. As a non-contact optical diagnostic technology, HSI can detect the distribution of lesions in body skin tissues, ex vivo cancer tissues, and organs such as eyes and teeth. It can play an important role in medical diagnostic scenarios such as cancer, heart diseases, retinal lesions, and dental diseases. In addition to medical diagnostic scenarios, HSI holds great promise for disease mechanism research and even surgery assistance. Compared with general imaging methods, HSI can acquire both image and spectral information, with the advantages of a wider range of bands, higher spectral resolution, and more comprehensive information acquisition. So, HSI can provide more accurate and dimensional information of the disease site in spatial and spectral dimensions. Not only can HSI obtain external information such as size and shape that can also be identified by other detection imaging methods, but HIS can also get the differentials of the internal structure. It also has a very high sensitivity. However, due to the effect of too much information and overlong processing time, it is difficult to achieve rapid diagnosis in real-time. Therefore, how to capture effective information quickly by fusing algorithms is one of the main research directions currently. In addition, there are few HSI devices and mass-produced products that are actually used for medical detection, and even the existing products are often very large in size and difficult to adapt to the needs of medical conditions. The main reason is that it is difficult to develop hyperspectral products for SWaP^3^-constrained platforms, which makes them inconvenient to be used in the actual diagnosis and surgery assistance. Therefore, developing SWaP^3^ HSI instruments with higher performance is an important direction for R&D in the field of medical devices.

## 3. Core Technologies of Intelligent SWaP^3^ Camera

With the great improvement of electronics, optics, materials science, artificial intelligence, and other related disciplines, many advanced technologies have been applied in the R&D of IRCs to achieve intelligence and SWaP^3^. This section analyzes the core technologies, including the realization of lightweight, miniaturization, intelligence, and high performance, including hyperspectral, high-resolution, HDR, and large FOV of intelligent SWaP^3^ Cameras.

### 3.1. Realization of Lightweight and Miniaturization

Achieving lightweight and miniaturization is the emphasis of intelligent SWaP^3^ camera R&D. Researchers need to deal with the contradiction among high-performance parameter requirements, volume, and mass. Furthermore, in space applications, they also need to consider the space environment, such as force, heat, and radiation. As mentioned above, the R&D level of detector chips plays a pivotal role in the lightweight of intelligent SWaP^3^ IRCs, which affects many aspects, including optical systems, cooling equipment, structures, materials, and electronic devices. Many research institutions and companies are constantly upgrading the level of detector development, namely by reducing the pixel size. Lior Shkedy et al. [59] used a 3-megapixel (1920 × 1536) XBn FPA-Hot (Higher Operating Temperatures) Blackbird array, which is a large-scale array with a small pixel pitch of 10 μm. The use of the Hot Blackbird array directly reduced the size and weight of the integrated detector cooler assembly (IDCA) and the optical system. Moreover, they reduced power consumption and cost and improved reliability while maintaining performance. Through the analysis of the above study, the R&D level of the detector directly affects the subsequent electronics design around it. In addition, as for optical systems, if they reduce the pixel pitch in the case of a certain number of pixels, the size and weight of the optical components and structures required for the system will be significantly reduced. What is more, the cooled equipment and components will also be lighter and more compact. Furthermore, with the cooling requirements reduced, it is possible to reduce the overall system power consumption. Finally, all the above-mentioned measures directly determine the cost. So, the design and R&D level of detector chips are particularly critical. However, while reducing the pixel pitch, the impacts on performance parameters such as QE should also be considered. So, in order to maintain the original performance or improve performance, it’s necessary to further optimize the design.

#### 3.1.1. Lightweight and Miniaturization of Optical Systems and Support Structures

**Figure 10:**
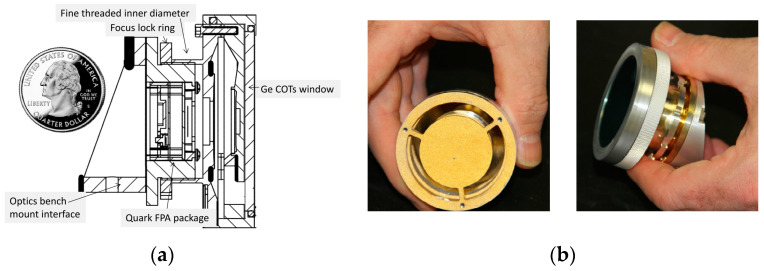
Comparison of the cross-sectional for the optomechanical engineering design and a quarter dollar coin [60]. (Reproduced with permission from SPIE; published by SPIE, 2014).

The optical components, optical systems, and their supporting structure are almost the largest part of the whole camera in terms of mass and space, so in order to achieve miniaturization and integration, the optimization of the optical system is a key part. Erin F. Fleet et al. applied folded path reflection and reflection optics. They used four concentric aspherical mirrors, aluminum plates, and the LWIR micro-radiometric thermometer array detectors to design and fabricate a compact LWIR imaging system that achieves performance close to the diffraction limit. Adopting the above measures, they achieved the miniaturization of the optical system and its structure [60]. In Figure 10a, a cross-section of the system’s optomechanical engineering design and a quarter-dollar coin are shown for comparison. Additionally, the lens system is about 15 mm long, and the whole system can reside within a 3-inch diameter ball gimbal. Thus, we speculated that the actual volume of the camera is less than 8.58 cm^3^ achieving a very high degree of miniaturization. The camera designed based on this LWIR optomechanical structure is shown in the figure, which supports our speculation. Figure 10b shows the LWIR optics (the left) and the entire camera (the right).

In terms of hyperspectral camera lightweight, due to the vastly improved micromechanics and microelectronics technologies, the spectral components, as the main part of the mass and volume, make great breakthroughs, which has contributed to the change in the spectral splitting method [61]. Moreover, the spectral components of hyperspectral imagers have been continuously developed in the direction of miniaturization and integration.

Due to the ability to adapt to external changes and maintain good working performance, adaptive optical systems [33] have further advanced the development of small-size, high-performance, and highly reliable cameras. In addition, the mechanical mechanism containing the optical system is often the biggest obstacle limiting the miniaturization of the camera. However, the optical system cannot be optimized at will. Furthermore, subject to the FOV, focal length, and other requirements, optical components and focusing mechanism also cannot be miniaturized optionally. So mechanical methods for achieving miniaturization are not desirable. In terms of optical systems, developers should carefully design and select suitable system types. Under the premise of meeting the requirements of the index, developers must adopt as few lenses and other accessories as possible. Moreover, researchers can use new optical systems and minimize the space occupied by the optical path. Furthermore, the support structure should also be optimized according to the optical system type. In summary, in order to determine the optimal solution, lightweight optical systems, and their structures often require a lot of experimental research in the design of structures and systems and the selection of materials.

#### 3.1.2. Integration of Electronics Systems

The integration of electronic systems is also an important means. Electronics miniaturization mainly involves three aspects: circuit design, electronic components selection, and the packaging process.

**Figure 11:**
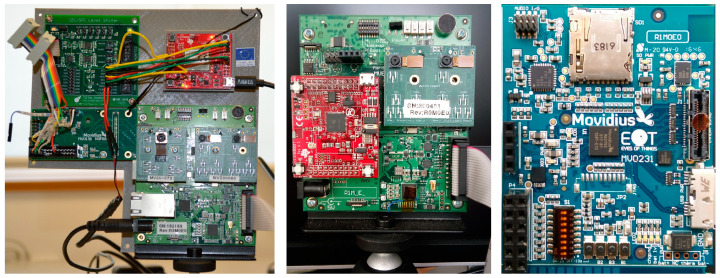
Iteration process of EoT Board size, from left to right (mm): 200 × 180, 100 × 100, 57 × 46 [62].

One of the means is to simplify the circuit design by reducing wiring and solder joints in the electronics structure to increase the integration. In the past, small and medium-sized integrated circuits were mostly used, but nowadays, developers mostly use large-scale integration (LSI) and very large-scale integration (VLSI). In addition, reducing unnecessary components and integrating additional external accessories into a single board is useful to simplify the circuit layout. In HyperScout 2 mentioned above [29,30], the CPU and EoT (Eyes of Things) Board [62]-an information processing and acceleration board-are equipped for data processing. The EOT developed by Oscar Deniz et al. can be considered a low-power, low-cost, and compact camera module. Figure 11 shows the optimization and iterations of the board three times from the prototype board to the final product. During the iterations, redundant components used very infrequently were removed, and external components were integrated into a single board, continuously reducing the cost, size, and power consumption of the submodule. In developing a new multi-mode camera [63], John Liobe et al. reduced the camera depth to 1/3 of the old model. They abandoned the traditional multi-board stack and adopted a single-piece rigid-flex assembly. Besides, they replaced the individually removable FPA package with a permanently bonded to the camera electronics direct-mound chip-on-board package. Moreover, the number of signals required for memory access was minimized by optimizing the memory interface structure thereby reducing the pin size and number of components required. Additionally, common power rails were used for the components to reduce corresponding power management circuitry. The aforementioned measures make it possible to miniaturize cameras, which can be references for R&D.

**Figure 12:**
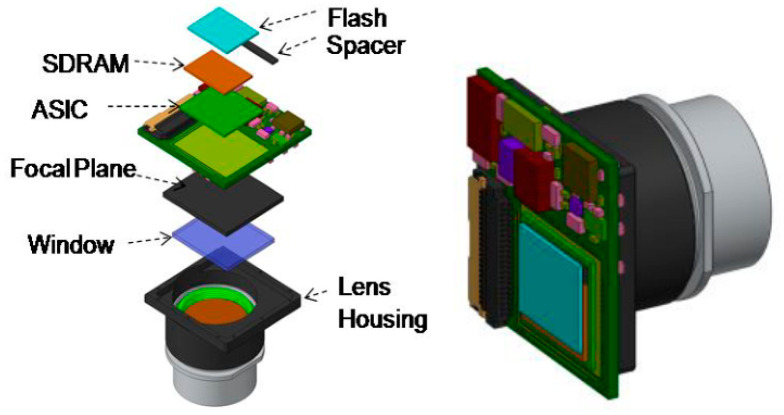
SMART chip camera model and distribution after disassembly of the dual panel [8]. (Reproduced with permission from SPIE; published by SPIE, 2015).

Then, as for the selection of electronic components, developers can use core components with higher integration and lower power consumption. Moreover, in order to ensure reliability, the adoption of mature, mass-produced, and large-scale-used devices and technologies is particularly critical. In general, DSPs and dedicated image processing chips usually take up relatively more resources, while FPGAs, ASICs, etc., can save resources and space. In a study by Louise Sengupta et al. of BAE systems [8], the approach used was to develop a double-sided circuit card assembly (CCA). As shown in Figure 12, images are captured on one side of the board through the focal plane array, optical system, and window, while image processing is performed on the other side through a 3-D stacked ASIC with memory chips, including SDRAM and Flash. Although more expensive than FPGAs, ASICs have obvious advantages for SWAP-constrained platforms.

Finally, the adoption of a new packaging process to reduce the size and weight of the circuit is also an important tool for the miniaturization of electronic systems. Packaging is an important part of the detector. Some of the designed products use multiple types of packaging for different scenarios. Two different types of packaging were designed by R. Fraenkel et al. [64]. One is a hermetically sealed ceramic package for commercial applications, and the other is an adhesively sealed “PCB” type substrate. The former is TEC-less, and the latter has TEC. Not only does it provide an ideal alternative SWaP solution for edge systems, but it also meets the environmental constraints required for some military-grade systems with low cost.

In addition, the development of camera systems tends to be modular. Currently, each module can be independently managed and controlled, further contributing to the development of miniaturization. Furthermore, the miniaturization of electronic systems requires comprehensive consideration of data processing, storage capacity design, power consumption control, and resource layout.

#### 3.1.3. Optimization of Power Management System

In addition to the two core means of optical and electronic systems mentioned above, the optimization of a power management system is also useful to achieve lightweight and integration. By adopting a new highly integrated power management system and equipping it with microprocessors, intelligent management, and rational layout can be realized. Developers can combine the idea of modular design and make full use of the shared area in the system, which is effective for optimizing the structure and reducing the complexity of the system.

**Figure 13:**
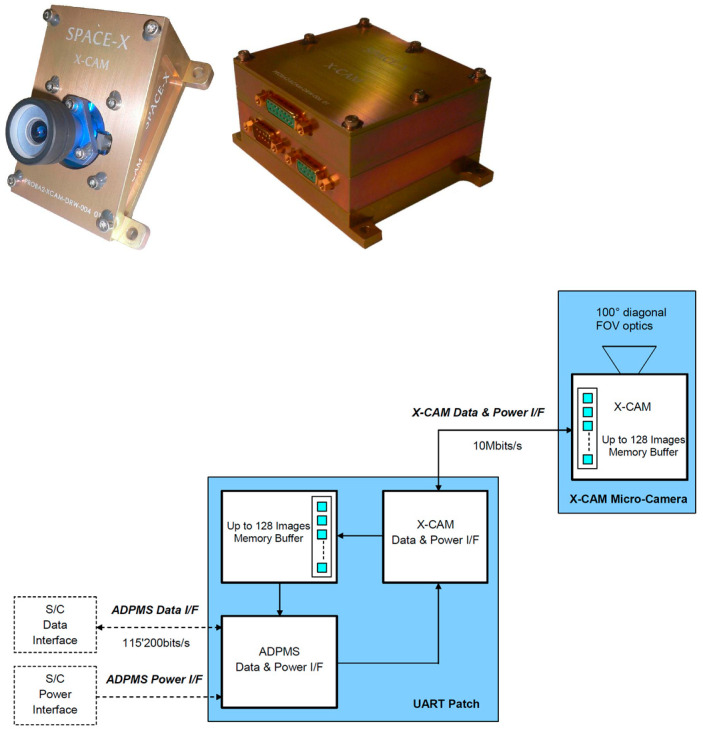
Physical diagram of X-CAM and its system structure schematic [66].

In ESA’s PROBE 2 program, as shown in Figure 13, the X-cam system it carries (which is in the visible band and can be used as a reference for IRCs’ power system design) [65,66] has a UART module apart from the camera module. The UART module is responsible for controlling the connection of the miniature camera to the spacecraft’s ADPMS (Advanced Data and Power Management System) for communication. Furthermore, the module consumes only 1 W of power, its weight is 413 g, and the volume is only 55 × 110 × 86 mm^3^.

#### 3.1.4. Application of New Materials

During the development of the SWaP^3^ IRCs, the selection of materials for the detector chip, electronic devices, optical components, and packaging has been a key factor affecting the cost and lightweight indexes of the camera. New composite materials are emerging, and the number of materials with different properties available for screening has increased greatly, which creates the conditions for lightweight.

New materials can not only bring weight advantages that traditional materials do not have but also further improve the stability of the whole system’s structure. In the case of materials for space cameras, the support structures of optical and other systems processed by conventional materials are more affected by temperature and humidity in the harsh space environment, which can affect the stability of camera performance or even interfere with normal imaging. The Shanghai Institute Of Ceramics, Chinese Academy Of Sciences has carried out years of R&D on silicon carbide matrix composites [67]. They solved the comprehensive coordination and balance of high stiffness, lightweight, and low thermal expansion of the optical support structures. What is more, the support structures and structural components developed have been successfully applied to the imaging loads of satellites such as the Gaofen-2 and some small commercial RS satellites.

#### 3.1.5. Improvement of Cooling Equipment (For Cooled IRCs)

In many applications, cooled IRCs have much higher performance than uncooled IRCs in terms of spatial resolution, sensitivity, spectral response, response time, and so on. The cooling equipment is the prerequisite for the proper operation of cooled IRCs. The traditional mechanical cooling equipment has the disadvantages of large size, poor durability, and low-cost performance, making it difficult to fulfill the requirements of SWaP^3^. Whereas TE systems are relatively lighter and smaller, and they have lower cost, as well as higher durability and reliability [68,69]. Therefore, it is very important to develop and optimize TE systems of cooled IR cameras. D. Crane et al. proposed a technique called Distributed Transport Properties, based on which the transport characteristics (Seebeck coefficient, resistivity, and thermal conductivity) of TE elements can vary with position, significantly improving the coefficient of performance (COP) and cooling power of TE systems [70]. Coupled with many advantages of the TE system itself, DTP has a broad future in many IRCs application scenarios, such as autonomous driving.

In addition, in the design process, considering the detector itself, increasing the upper limit of the normal operating temperature of the IRFPA as much as possible [59] can reduce the heat dissipation requirements of the system. Then, the need for cooling equipment and components can be reduced to decrease the size and weight of the whole machine.

In summary, there are many ways to realize the lightweight and miniaturization of IRCs, and different measures can be taken for specific requirements. However, it should be noted that the reliability of the camera system is often irreconcilable with miniaturization, so the most appropriate balance must be maintained. Furthermore, it is also necessary to deal with the contradiction among the high resolution, large FOV optical system, size, and weight. In the resource-oriented design, the performance can be appropriately sacrificed according to the scene requirements to achieve miniaturization and lightweight. However, in most scenarios, miniaturization must not be achieved at the cost of reliability. In order to improve more elements, all aspects of SWaP^3^ need to be addressed in a balanced manner.

### 3.2. Implementation of High Performance 

#### 3.2.1. Hyperspectral Technology

For the hyperspectral SWaP^3^ IRCs, the implementation of hyperspectral technology is vital. Moreover, fine spectroscopic technology is the key to achieving HSI, and it is also the main distinction among single-band, multispectral, and HSI. So it is important to research the ways to achieve hyperspectral [71].

According to different spectroscopic methods, the mainstream types of hyperspectral mainly include grating spectroscopy, interferometric spectroscopy, prism spectroscopy, and AOTF (acousto–optical tunable filter spectroscopy). In space applications, the hyperspectral imagers take dispersive spectroscopy (grating, prism) and interferometric spectroscopy (Fourier transform). There are also some cutting-edge technologies such as LCTF (liquid crystal tunable filter), chip coating, micro-integrated gradient filters, thin-film optics, binary optics, and Farber cavity MEMS chips [71]. The mainstream technologies are not further discussed, and the focus in this part is on the introduction and analysis of the advanced means of hyperspectral technology.

AOTF, whose full name is acousto–optic tunable filter, consists of an acousto–optic medium, a transducer, and an acoustic terminal. RF drive signal through the transducer in the acousto–optic medium to excite the ultrasonic waves. Changing the frequency of the RF drive signal changes the wavelength of the diffracted light of the AOTF, thus enabling electrically tuned wavelength scanning [72,73]. The technology is implemented in a relatively simple way, but it has certain drawbacks; not possible to achieve a large size. So, the performance will be limited accordingly, and it is difficult to apply in scenarios with high spectral resolution requirements. As for liquid crystal tunable filter (LCTF), applying voltage can change the orientation of the liquid crystal molecule arrangement, then using the electronically controlled birefringence effect can modulate the phase difference; at last, the different wavelength of light interfere with achieving continuous tunability scanning [74]. HSI equipment developed based on LCTF has the characteristics of small size, low weight, and low power consumption, so it is very suitable for SWaP^3^-constrained platforms. However, the liquid crystal itself is very sensitive to the external ambient temperature, so it is easy to cause temperature drift to make the detection results inaccurate. In addition, according to the products developed so far, the cost is high. AOTF and LCTF are both tuning spectroscopic means [75], which are suitable for light miniaturized platforms.

Not needing a traditional beam-splitting module, Chip coating technology only requires the coating of filter films in different wavelengths on the detector pixels to an image in tens or hundreds of spectral bands within a set range. Therefore, Chip coating reduces the number of spectroscopic components and greatly simplifies the beam-splitting structure and the support structure, further increasing the degree of integration. IMEC (Interuniversity Microelectronics Centre) has conducted much research in this area and has used highly sensitive CCDs and sCMOS chips for development to achieve HSI [76,77], which provides a new solution for the development of lightweight and compact hyperspectral cameras. However, due to directly coating on the chip, it requires a high production process. Moreover, it cannot be continuously adjustable in the full spectrum, thus lacking flexibility and having limitations in applications. In addition, the use of line array filter elements and pixel-level multi-channel filters for HSI also achieves lightweight by simplifying the spectroscopic structure.

Another fused advanced technology is the Fabry–Perot cavity MEMS chip. It is based on the principle [78,79] of using a Faber cavity to complete different spectral image information acquisition through fast broad spectral input and specific spectral selective output, i.e., using a time-modulation method for spectral modulation to achieve HSI. The Fabry–Perot cavity MEMS chip incorporates the microfabrication process of MEMS devices and mature detector chip development technology to provide a small size, high stability, and cost-effective hyperspectral solution but with a high technical threshold.

**Figure 14:**
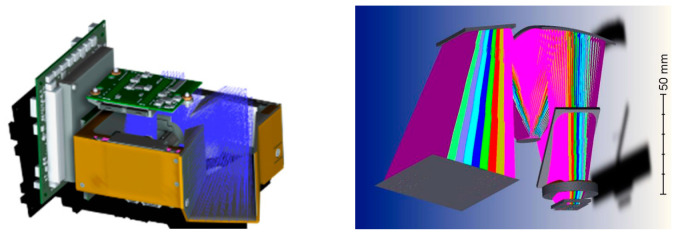
Schematic diagram of HyperScout 2’s optical path structure [29,31].(The color lines in the figure indicate the optical layout).

Moreover, there are studies that have achieved dual-channel HSI; each channel uses a different means of spectroscopy. HyperScout 2 uses a reflective TMA (off-axis three-mirror astigmatic) based on free-form design and diamond-turning machining [29], which is shared by the VNIR and TIR channel. As shown in Figure 14, a beam splitter is inserted at the end of the optical chain, and the shorter wavelength turns 90 degrees to the VNIR sensor, and the longer wavelength impacts the TIR sensor, thus achieving a dual channel. The dual-channel solution makes it the first onboard optical payload to share a single optical path in both wavelength ranges. Furthermore, the hyperspectral realization for each wavelength band is as follows: In the VNIR band, HyperScout 2 uses a linear variable hyperspectral filter to separate waves in different wavelengths, while in the TIR band, a set of four spectral filters to achieve multispectral are used.

#### 3.2.2. HDR, Large FOV, High Resolution, DW, and High Frame Rate

HDR is mostly achieved by algorithms. I. Hirsh et al. [80] performed algorithm optimization for SWIR images with HDR. In order to increase the limited DR of the detector, a dedicated algorithm based on two exposures was developed. Through this algorithm, cameras can capture two consecutive frames with different exposure times and fuse the two frames into a single frame with an extended dynamic range. Moreover, the imager also has other image processing algorithms, including NUC and dynamic range compression optimized for low light level (LLL) conditions. In addition, the camera measures 31 × 31 × 32 mm^3^, weighs 50 g, and consumes less than 1.4 W, making it a typical SWIR SWaP^3^ camera.

In terms of large FOV, firstly, taking a large-scale array detector is the most direct and effective way. Dali Technology company has developed a 6-megapixel uncooled IRFPA detector with a 3072 × 2048 array size conducive to providing high-speed, stable, uniform, and detailed infrared images, realizing the demand for large FOV and wide dynamics. Furthermore, it has a wide application prospect in RS, situation awareness, and other aerospace applications. However, enlarging FOV by taking large-scale array detectors is subject to process and cost constraints, and it also has physical limits. Additionally, optimizing the optical system, fusing multiple sensors, and performing field-of-view stitching are all effective means to enlarge the FOV of camera systems [81,82,83].

High resolution is often largely determined by the detector chip. Furthermore, the detector is very small compared to the whole machine, so it has little effect on the miniaturization and lightweight of the camera. Therefore, the primary means to achieve high resolution is to start from the size of the detector. Moreover, as mentioned above, the optimization of the optical system can also further improve the resolution. In addition, super-resolution algorithms and image enhancement algorithms can also be used to improve image resolution [84,85,86].

The dynamic window opening technique can improve the frame rate by reducing the imaging area during detector readout. In the development of the landing camera of Chang’e-2 APS (Advanced Photo System) [10,87], BISME (Beijing Institute of Space Mechanics & Electricity) realized two modes on it: high resolution (whole-frame output) and high frame rate (open-window subsampling). In the former mode, the effective number of pixels is 1280 × 1024, and the frame rate is 1 fps. In the latter mode, 640 × 512 pixels are selected from 1280 × 1024 pixels, and the frame rate becomes 10 fps. The working mode can be controlled by programmable commands. Moreover, the camera has integrated intelligent operations such as automatic shooting and real-time image compression. Additionally, it is highly reliable in harsh space environments. The APS also has three surveillance cameras in the visible band, which are also typical examples of SWaP^3^ cameras.

To sum up, there are abundant technical ways to improve each performance parameter, which can be selected with specific requirements. However, it should be noted that the FOV and resolution cannot be improved simultaneously. So, in the case of resolution and optical system aperture in check and balance, how to solve the contradiction, namely both to achieve the purpose of high-resolution information acquisition and large FOV reconnaissance, is a difficult but vital task to solve currently. The optical research in this area mainly focuses on the stitching of detectors, lenses, and images to achieve large-field-of-view and high-resolution systems. Researchers concentrate on the following four aspects: multiple small-scale detector stitching, single high-resolution lens stitching, multiple high-resolution lenses taking and stitching images simultaneously, and multi-scale optical systems [35,81]. Additionally, the large field-of-view optical systems are also contradictory to integration and miniaturization. All in all, rational and comprehensive design are crucial.

### 3.3. Realization of Intelligence

The realization of intelligent acquisition, processing, and interaction of IRCs is closely related to the development of advanced technologies, including computer networks, artificial intelligence, edge computing, cloud computing technology, embedded, etc. From the perspective of hardware, the integration of GPU, VPU, and other processors with high-speed real-time information processing capabilities is adopted to achieve intelligence. In terms of software and algorithms, developers often deploy image pre-processing, autofocus, image compression, target detection and identification, and many other intelligent algorithms on cameras. Furthermore, in the user terminal, the R&D of supporting intelligent and convenient software is helpful for human–computer interaction.

### 3.4. Achievement of Low Power Consumption

There are a few ways to achieve low power consumption. Just like the Banpil camera that discards additional components through on-chip AD technology [23], in the R&D of the intelligent SWaP^3^ IRCs, replacing the more complex and power-consuming parts with relatively easier and lower power-consuming accessories is one of the important ways to achieve low power consumption. Replacing and updating by using new, low-power, mid-range performance processors and storage devices make it more suitable for the needs of intelligent compact cameras.

In the R&D of a multi-mode tracking (MMT) camera, a team of SUI [63] took measures of changing active cooling by TEC to passive cooling to reduce overall power consumption. Moreover, they developed a temperature-based non-uniformity correction algorithm to compensate for the problems associated with the cooling method change. In these studies [23,48,63], low power consumption was achieved by directly removing the component, which is the main source of power consumption. Then the negative effects of this means were usually corrected by changing the circuit structure or developing corresponding compensation algorithms frequently used. 

## 4. Development Tendency of Intelligent SWaP^3^ Camera

In the above, we analyze the development and core technologies of intelligent SWaP^3^ IRCs in terms of lightweight, miniaturization, low cost, low power consumption, intelligence, and high performance, including hyperspectral, high resolution, large FOV, and HDR. What is more, we also briefly explain the primary applications of intelligent SWaP^3^ IRCs. By summarizing the above works, we propose the following directions for the development of SWaP^3^ cameras in the future.

### 4.1. Develop Chips with Smaller Pixel Pitch and Larger Scale Array Size

Focus on the development of smaller pixel size and larger array size detector chips. The development level of the detector chip is the top priority of intelligent SWaP^3^ camera R&D, which determines the upper limit of intelligent SWaP^3^ IRCs in terms of performance. The core parameters of a detector chip, including pixel pitch, array size, response rate, sensitivity, NETD, operating temperature, response time, etc., have an impact on the design of various aspects of cameras comprising the optical systems design, electronics systems layout, materials selection and so on. Furthermore, as we have mentioned in the previous section, all aspects of SWaP^3,^ including size, weight, performance, power consumption, and price, are affected by the camera’s components, namely revolve around the detector chip.

### 4.2. Broaden the Resolution in Multi-Dimension

Broaden the multidimensional resolution of the camera, i.e., improve the spatial resolution and spectral resolution. Furthermore, for space RS applications, temporal resolution is also considered. In terms of spatial resolution, the increasing development level of the detector’s pixel size and array scale and new optimization algorithms improve the spatial resolution of images continuously, thus enabling the camera to capture more detailed information. In terms of spectral resolution, the division of spectral bands is finer and finer, and the number of bands becomes more and more. Then the camera system can identify and distinguish more types of targets. However, the spectral resolution and spatial resolution are mutually constrained, and the simultaneous improvement of both will reduce the signal-to-noise ratio and affect the imaging quality. So, a reasonable balance has to be found. In addition, for applications of RS, it is also necessary to pay attention to the time resolution, the concept of which is the minimum time interval between two observations of the same area. Furthermore, it indicates the frequency of repeated observations, namely revisiting a time in applications. The shorter the interval and time, the higher the time resolution and the more time to observe clearly. So, high revisit time is helpful for achieving dynamic real-time observation. With technological breakthroughs, constructing constellations in a multi-star network is gradually applied, which can improve the time resolution for a high revisit to achieve all-weather real-time observation compared to single-star observation. However, the above-mentioned multi-dimensional resolution cannot be increased indefinitely. The increase in resolution will increase the amount of data extremely significantly, which will be limited by the data acquisition, transmission, processing, and storage capacity. A large amount of data may lead to an increase in errors and also cause some confusion. Not only will the excess data affect the acquisition and processing of image information that determines the final imaging performance, but it also undoubtedly increases the possibility of data errors due to the limitations of communication capabilities in the transmission of data. So, under certain limits, it’s necessary to do reasonable and effective allocation and promotion according to the scenario.

### 4.3. Embed Cutting-Edge Technologies and Deploy Intelligent Algorithms

One of the vital directions of developing and optimizing intelligent SWaP^3^ cameras is to embed various frontier technologies and deploy AI algorithms. Furthermore, combining emerging technologies such as neural networks and machine learning with mature image acquisition and processing technologies is also effective. In the process of camera design, apply large-scale array, small pixel pitch FPA, image stitching, sensors fusion, and other technologies to promote the lightweight, high-performance, and intelligent enhancement of the camera. Moreover, developers can try to integrate GPU, VPU, and other intelligent processing units into the core processing module. Then to enhance the intelligence of information perception and acquisition and data processing, researchers can deploy intelligent algorithms such as image pre-processing, lossless compression, image stitching, target detection, auto-focusing, etc., on the processor chips and achieve algorithms acceleration via intelligent hardware.

### 4.4. Build Multi-Camera Systems by Multi-Sensors Fusion and Optimization

Another trend in the development of intelligent SWaP^3^ cameras is multi-sensor or multi-camera fusion. The information that can be obtained by a single functional type of camera is limited, but by integrating two or more types of imaging devices and performing data fusion processing, image, spectrum, and polarization information can be obtained in a unified manner. In this way, the information interoperability between devices can be increased, thus improving the reliability of the system and enhancing information utilization, comprehensiveness, science, and coverage to achieve broader situational awareness. Multi-sensor fusion technology has been applied in many fields, such as medical care and autonomous driving [88,89,90]. In the medical field, for example, Kai Lin et al. [88] developed a hybrid body sensor network architecture based on multi-sensor fusion (HBMF), which can be used in medical robots to perform simple diagnoses and treatment to reduce physicians’ workload. It combines various sensors (various biomedical devices inside or on the surface of the human body, wearable devices, etc.) and processors to achieve intelligent medical care. In many medical human–computer interaction (HCI) scenarios, including assisted diagnosis, surgical assistance, and nursing patients, the HBMF intelligent system can obtain multiple data sources of users and environment, which is more diverse, flexible, and objective and ensures the reliability of machine diagnosis and treatment. Moreover, optimization refers to optimizing existing camera systems with emerging intelligent IR lightweight devices. The aforementioned measures can further achieve the purpose of improving the performance, increasing the functionality, and widening the scenario.

### 4.5. Extend the Concept of SWaP^3^

Reliability, stability, extensibility, safety, etc., are important factors that must be considered in applications. Reliability indicates the ability of the camera system or its components of it to work normally without failure in a certain period of time and under certain conditions, which is a prerequisite for use. The stability of the camera system can be considered as the degree of stability of performance for working in a certain period of time and under certain conditions. Therefore, if the core performance of a camera system is good or bad, then it lacks stability. Extensibility is also important for the camera system. As for hardware, extensibility means enhanced scalability within the limited space layout and weight restrictions, i.e., increase the interface to expand the module. In addition, modularity is also an important means to achieve hardware expandability, which achieves expansion by means of easy replacement of the same type of modules, hot-swapping, and so on; from the software perspective, extensibility refers to the ability to add new functions or improve existing functions to adapt to future development. Furthermore, the independence and compatibility with hardware are also a reflection of the software’s extensibility. Security means the nature of the system to work properly without external intrusion, which often refers to the security of the network and information. The above-mentioned elements are more or less in a mutually facilitating or inhibiting relationship with the core elements of size, weight, and performance already present in SWaP^3^. Therefore, broadening the concept of SWaP^3^ and considering more concerns in applications can make the camera systems more comprehensive, practical, and efficient.

The development tendency proposed in this paper is to achieve the ultimate goal-reduce the size, weight, power consumption, and price while improving the performance and enhancing the reliability, stability, and scalability of IRCs. There is no doubt that continuously proposing new principles and solutions and developing new means to achieve SWaP^3^ is very important. However, it is even more vital to do a good job of coordination in the system design. Developers should start from the overall resource allocation of the system and take into account all aspects. Last but not least, developers should not pursue a single performance optimum while causing a waste of resources, let alone improve performance, reduce cost and achieve lightweight at the expense of reliability and stability.

## 5. Conclusions

This review mainly focuses on the development of intelligent SWaP^3^ IRCs, in which we analyze the development of various camera indicators, including size, weight, performance, power, price, and intelligence. Then, we discuss the core technologies involved in detail. In the past, many tools have been adopted to develop intelligent SWaP^3^ cameras and optimize the various elements involved in SWaP^3^, but there are still some difficult problems and challenges. Therefore, by analyzing and summarizing the current development status, researching technology approaches, current problems, and pain points in recent years, we summarize the present status and propose several key development directions for intelligent SWaP^3^ cameras in the future. It is necessary to continuously expand the concept of the SWaP^3^ and improve the whole machine from multiple angles and levels, such as the chip, camera, and system. It is hoped that this paper can provide a reference for the R&D of intelligent SWaP^3^ IRCs and broaden SWaP^3^ IRCs’ application scenarios in more fields in the future.

## Figures and Tables

**Table 1:** Technical indicators and characteristics of typical intelligent SWaP^3^ infrared cameras (IRCs) around the world in recent years.

Development/ On-Board Deployment Time	Camera Name	Institution/ Company	Band	Scenes	Size, Weight, Power	Solution	Cooled or Not (Y/N)	Highlighting Technology
2010	Chang’e-2 Advanced Photo System’s (APS) landing camera [9]	Beijing Institute of Space Mechanics & Electricity (BISME)	VNIR	Satellite surface imaging	Size: 100 × 80 × 81 mm^3^ Weight: 502 g Power: 4 W	1280 × 1024 640 × 512	N	Dual mode: High resolution and high frame rate mode
2011	ATOM1024 XGA	Sofradr EC	LWIR	Large FOV, situation awareness, surveillance	Size: 61 × 69 × 66 mm^3^ Weight: <110 g Power: <3.7 W	1024 × 768	N	Uncooled micro-radiometer camera wide detection range
2013	Tamarisk 320 Cameras	DRS	LWIR	High sensitivity and high image quality requirements	Size: <34 × 30 × 30 mm^3^ Weight: <37 g Power: <1.1 W	320 × 240	N	With non-uniform correction, real-time video processing
2013	Tamarisk 640 Cameras	DRS	LWIR	High sensitivity and high image quality requirements	Size: 46 × 40 × 31 mm^3^ Weight: <73 g Power: <1.5 W	640 × 480	N	With non-uniform correction, real-time video processing
2014	Banpil Camera	Banpil Photonics	VIS-SWIR Multispectral	Identification and detection in all weather and visibility conditions	Size: Not mentioned Weight: Not mentioned Power: <1 W	640 × 512	N	Low dark current Multispectral imaging High-performance FPA HDR
2015	SMART Chip Camera	BAE Systems	LWIR	Edge scenarios such as handheld	Size: 2.9 cm^3^ Weight: 5.1 g Power: <0.5 mW	640 × 480 1280 × 1024	N	“SMART” Architecture Android GUI
2016	TEC-less SWIR Camera	UTC Aerospace Systems	SWIR	Various scenarios requiring SWIR	Size: Not mentioned Weight: Not mentioned Power: <1.4 W	1280 × 1024	N	no TEC Current mirror-type pixel
2016	Hot Blackbird XBn detector	SCD	MWIR	Surveillance, missile warning	Size: Not mentioned Weight: 730 g Power: 7.5 W	1920 × 1536	Y	10 μm small pixel size Smaller cooled device
2018	SWIR Imager based on Cardinal 640	SCD	SWIR	Day and night imaging: strong daylight and LLL	Size: 31 × 31 × 32 mm^3^ Weight: 50 g Power: <1.3 W	640 × 512	Y	Video engine image processing algorithm HDR LLL High Adaptation
2018	FLIR Tau 2+ [10]	TELEDYNE FLIR	LWIR	In-orbit observation	Size: 82 × 82 × 145.08 mm^3^ Weight: 422.8 g Power: <1.2 W	640 × 512	N	High sensitivity Multiple video formats
2018	CIRES cameras [10]	TELEDYNE e2v	VNIR	In-orbit observation	Size: 40.8 × 36.6 × 34.1 mm^3^ Weight: 70 g Power: 1.5 W	1280 × 1024	N	Multi-image processing algorithms High frame rate
2018	MCAMv3 camera	microcameras.space	VNIR	In-orbit monitoring on solar panels and critical loads	Size: 63.1 × 45 × 85 mm^3^ Weight: 327 g Power: <1.8 W	4 million	N	Embedded image compression High Resolution HDR
2020	HyperScout 2	Cosine	VNIR/LWIR Hyperspectral	Fire detection, heat island effect, etc.	Size: 1.8 L Weight: 1.8 kg Power: 11 W	4000 × 1850/1024 × 768	N	VNIR/TIR Dual channel Hyperspectral/AI Chip BEE/OBDH Subsystem
2021	Griffin-5 HOT MWIR	Attollo Engineering	MWIR	MWIR edge scenario	Size: 166 cm^3^ Weight: 250 g Power: <4 W Power with cooling: <10 W	640 × 512	Y	5 μm small pixel pitch sensor
2022	HyperScout M	Cosine	VNIR Hyperspectral	Fire detection, heat island effect, etc.	Size: 1.2 L Weight: 1.2 kg Power: 9 W	4096 × 1850	N	Hyperspectral AI Chip BEE/OBDH Subsystem
2022	Griffin-8 HD	Attollo Engineering	SWIR/MWIR dual Band	SWIR and MWIR edge scenarios	Size: 97 × 50 × 50 mm^3^ Weight: <250 g Power: <6 W Power with cooling: <10 W	1280 × 720	Y	8 μm small pixel pitch sensor
2022	Multi-Mode Tracking (MMT) Camera	Collins Aerospace	SWIR	Multimodal scenes	Size: 25 × 25 × 7 mm^3^ Weight: Not mentioned Power: 1.5 W	1280 × 1024	Not mentioned	Multimode: Support passive and ALPD active imaging

**Table 2:** Main materials used by detector development companies.

Company	Materials
FLIR	InGaAs/InSb
DRS	VOx
BAE	InGaAs/InSb
SCD	HgCdTe/InSb
Sofradir	VOx
NEC	HgCdTe
Teledyne Imaging Sensors	HgCdTe
Raytheon Vision Systems	Vox/HgCdTe
ULIS	HgCdTe
Selex	HgCdTe
AIM	InGaAs
Goodrich Corporation	VOx

**Table 3:** Core parameters of typical HSI instruments applied and under research in recent years around the world.

Time	Instrument Name	Band Range/μm	Bands	Spectral Resolution	Spectroscopic Method
2009	Hyper-Cam	MWIR: 3–5/LWIR: 8–11.8	256	<0.25 cm^−1^	Fourier interference spectroscopy
2009	HTV/HREP-HICO	0.35–1.08	128	5.7 nm	Not mentioned
2010	MAKO	7.8–13.4	128	47 nm	Grating splitting
2011	MAGI	7.1–12.7	32	100 nm	Grating splitting
2011	Tiangong-1	0.4–2.5	130	10/23 nm	Prism splitting
2012	AVIRIS-NG	0.38–2.51	425	8.1 nm	Grating splitting
2016	HyTES	7.5–12.0	256	18 nm	Grating splitting
2016	Resurs-P3/GSA	0.4–1.1	216	5–10 nm	Not mentioned
2016	MAKO-Updated	7.8–13.4	128	44 nm	Grating splitting
2016	ATHIS	8–12.5	155	38 nm	Grating splitting
2018	HyperScout 1	0.45–0.95	50/Up to 120 in boost mode	16 nm	Line array filter element
2018	DESIS	0.4–1.0	235	2.55 nm	Grating splitting
2018/2020	GF-5/GF-5-02	0.45–2.5	330	5/10 nm	Grating splitting
2019	PRISMA	0.4–2.5	238	12 nm	Prism splitting
2019/2021	ZY-1-02D/ZY-1-02E	0.4–2.5	166	10/20 nm	Grating splitting
2020	HISUI	0.4–2.5	185	10/12.5 nm	Grating splitting
2020	SIHIS	MWIR: 3–5/LWIR: 8–12.5	MWIR: 167/LWIR: 163	MWIR: 30 nm LWIR: 50 nm	Grating splitting
2020	EnMAP	VNIR: 0.42–1.00 SWIR: 0.9–2.45	228 (VNIR up to 99/SWIR up to 163)	VNIR: 6.5 nm SWIR: 10 nm	Prism splitting
2020	HJ-2	0.45–2.5	215	4.5/14 nm	Fourier interference spectroscopy
2020	HyperScout 2	VNIR: 0.45–0.95 LWIR: 8–14	VNIR: 50 LWIR: 3	16 nm	Unique single optical path dual channel and line array filter element
2020	HYPXIM CA	0.4–2.5	>200	10 nm	Prism splitting
2021	SHALOM	0.4–2.7	241	10 nm	Grating splitting
2022	HyperScout M	0.45–0.95	50/Up to 120 in boost mode	16 nm	Line array filter element

## Data Availability

Not applicable.
